# Discrimination of Oil Slicks and Lookalikes in Polarimetric SAR Images Using CNN

**DOI:** 10.3390/s17081837

**Published:** 2017-08-09

**Authors:** Hao Guo, Danni Wu, Jubai An

**Affiliations:** Information Science and Technology College, Dalian Maritime University, Dalian 116026, China; 1120150307_wdn@dlmu.edu.cn (D.W.); jubaian@dlmu.edu.cn (J.A.)

**Keywords:** Synthetic Aperture Radar (SAR), pattern recognition, oil slicks, lookalikes, feature fusion, Convolutional Neural Network (CNN)

## Abstract

Oil slicks and lookalikes (e.g., plant oil and oil emulsion) all appear as dark areas in polarimetric Synthetic Aperture Radar (SAR) images and are highly heterogeneous, so it is very difficult to use a single feature that can allow classification of dark objects in polarimetric SAR images as oil slicks or lookalikes. We established multi-feature fusion to support the discrimination of oil slicks and lookalikes. In the paper, simple discrimination analysis is used to rationalize a preferred features subset. The features analyzed include entropy, alpha, and Single-bounce Eigenvalue Relative Difference (SERD) in the C-band polarimetric mode. We also propose a novel SAR image discrimination method for oil slicks and lookalikes based on Convolutional Neural Network (CNN). The regions of interest are selected as the training and testing samples for CNN on the three kinds of polarimetric feature images. The proposed method is applied to a training data set of 5400 samples, including 1800 crude oil, 1800 plant oil, and 1800 oil emulsion samples. In the end, the effectiveness of the method is demonstrated through the analysis of some experimental results. The classification accuracy obtained using 900 samples of test data is 91.33%. It is here observed that the proposed method not only can accurately identify the dark spots on SAR images but also verify the ability of the proposed algorithm to classify unstructured features.

## 1. Introduction

Marine oil slicks occur during the extraction and transportation of crude oil. The development of marine transportation and ocean development technologies has increased the possibility of oil accidents. The high frequency of oil slicks at sea has not only caused a serious waste of energy but also seriously damaged the marine ecology and environment. In order to prevent oil slick disasters, it is essential to detect the location of oil slicks. Synthetic Aperture Radar (SAR) sensors can be operated day and night under all weather conditions and produce high-resolution images [1].

In recent years, the application of SAR in oil slicks monitoring has been improved thanks to the use of launched polarimetric SAR missions, such as Radarsat-2, ALOS-2 and so on [2]. Damping the Bragg scattering from the ocean surface is the basic underlying principle of SAR oil slick detection, and they produce dark spots on SAR images [3]. However, several phenomena (e.g., plant oil, oil emulsion, etc.) also produce dark regions in SAR images. These are called lookalikes and appear quite similar to oil slicks. These lookalikes often become false positives in oil slick detection. Many studies into discriminating oil slicks from lookalikes have been conducted with a wide variety of methods. These can be mainly divided into the following two categories. The first is methods that use specific classification features. Zheng Honglei et al. used polarimetric characteristic Single-bounce Eigenvalue Relative Difference (SERD) for oil slick detection and found that SERD can distinguish plant oil from crude oil relatively well [1,4,5,6,7,8]. Bing Duan et al. proposed a method based on cross-polarization ratio of multi-polarimetric SAR images to distinguish mineral oil from plant oil [9,10]. Yongsheng Yang et al. have used texture features to detect and classify oil slicks in SAR images [11,12,13,14]. Reliance on a single feature to distinguish oil slicks from and lookalikes limits the accuracy of conventional oil slick services based on single polarimetric SAR imagery [15]. The texture features of single polarimetric SAR images cannot fully describe the physical characteristics of the sea surface targets, which may cause misjudgment during the oil slick detection [1]. In consideration of the complementarity among features to the classification performance, multi-feature fusion would be more suitable for discrimination oil slicks from lookalikes. Multi-feature fusion is known as early fusion, and it captures all the underlying statistical information about the problem [16]. It may be possible to use multiple features simultaneously to reduce the false positive rate in quad-polarimetric SAR images.

The next goal is to improve the classification algorithm. Suman Singha et al. have presented a new oil slick classification system using Artificial Neural Networks (ANN) in sequence for image segmentation and feature classification [17,18,19]. Kruti Vyas et al. used hysteresis algorithms to segment the dark spots and decision trees to classify oil spills from lookalikes [19,20,21,22,23,24]. ANN has many advantages, such as high accuracy, strong ability of parallel distributed processing and good descriptiveness of nonlinear relationship between input and output [17]. However, the number of parameters in the input and hidden layers grows with the number of features used for classification (multiple input parameters), hence making the network training a very challenging task [25]. Sometimes the increase in the number of parameters can draw out the ANN’s learning time, and it may fail to achieve the purpose of learning [25]. In the decision tree, information gain tends to be biased towards features which have greater values. The decision tree method can lead to the emergence of overfitting problems, and it disregards the correlation between attributes of data sets [25]. Convolutional Neural Network (CNN) is a deep learning method specially designed for image classification and recognition [26,27,28,29,30,31]. It is designed to resemble multilayer neural networks. CNN is good at recognizing two-dimensional shapes and learning structural features and it provides a way to discern features from pixels automatically [32]. The short training time makes it easier to use multilayer neural networks and improves the recognition accuracy. Its successful use in image classification and recognition has shown that CNN produces satisfactory results in hand-written recognition [32]. CNN is also used in image segmentation [33,34,35,36,37]. Adhish Prasoon et al. have used CNN to perform the segmentation of knee joint cartilage in Magnetic Resonance Imaging (MRI). Its accuracy is much greater than that of the traditional method, and the training time was shorter [38].

In this paper, an algorithm is presented for discriminating oil slicks from lookalikes in SAR images. This algorithm is based on CNN and multi-feature fusion. The proposed algorithm is run in the following steps: detection of dark spots in SAR images, extraction of features, analysis and selection of features, selection of Regions Of Interest (ROI) in feature images, and classification of dark spots into oil slicks or lookalikes. During feature extraction, 12 different kinds of features consisting of 4 polarimetric features and 8 texture features are extracted. During feature analysis and selection, three features are used to produce the desired feature subset, which has good distinction. In the classification step, CNN model was built and verified through three data. Training data set of 5400 samples serves as the input to train the CNN, and the classification accuracy obtained using 900 samples of test data is 91.33%. The ANN classification accuracy was only 80.33% for the same test data. In addition, dark spots of all five experimental data were predicted by the established model of CNN to confirm our result. The proposed algorithm can effectively apply CNN to the classification of oil slicks and lookalikes in SAR images. The learning sample of CNN is the ROI selected on dark spots of the feature images, which does not have any characteristic shape. The experiment verified that the proposed algorithm is also good at learning non-structural features.

The rest of this paper is organized as follows. In Section 2, five quad-polarimetric SAR oil slick scenes acquired by C-band Radarsat-2 polarimetric mode are introduced. In the third section, the features of the dark spots are extracted and analyzed, and then an optimal feature subset based on feature fusion is constructed to identify the oil slicks and lookalikes. In the fourth section, we describe the classification based on CNN, in which the learning process is based on the feature values. The effectiveness of the algorithm is demonstrated through the analysis of some experimental results and the results are compared to those produced using ANN. The conclusion and outlook are discussed in the final section.

## 2. Image Data Sets 

Five quad-polarimetric SAR oil slick scenes were acquired by C-band Radarsat-2 polarimetric mode. The first three sets of data (No. 1~No. 3) are used for the establishment and validation of CNN model, which contain training and test data. The last two data (No. 4 and No. 5) are mainly used for the prediction of the established model CNN, which contain test data only. The polarimetric SAR data are further described in Table 1.

### 2.1. Mexico Bay Area Data Set (No. 1)

**Figure 1:**
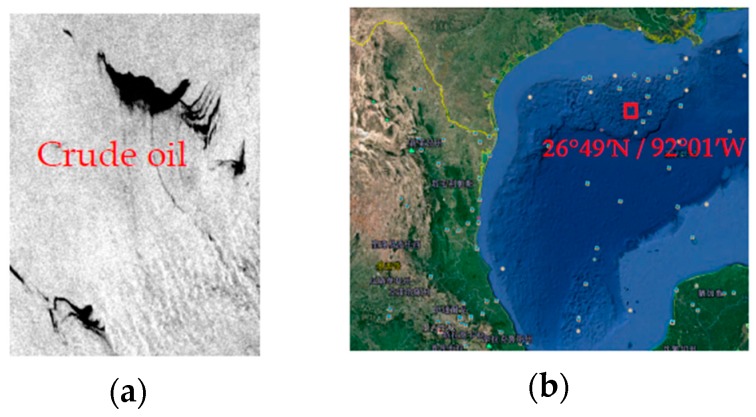
Radarsat-2 scene on 8 May 2010 in the Mexico Bay area. (**a**) The gray scale of the oil slick data in the Mexico Bay area; (**b**) The location of the incident.

The first experimental data is an area in the Mexico Bay. The scene was captured in 8 May 2010. Explosion of the offshore drilling platform lead to the oil slick. The dark spots in the image have been interpreted as crude oil [39]. The oil slick covers an area of 160 km; the width of the widest range is 72 km. The wind speed and wind direction were about 6.5 m/s and 167° [39]. Because the Gulf of Mexico in May is the end of spring and the beginning of summer, the water temperature is about 20 °C. The gray scale of the oil slick data in the Mexico Bay area and the location of the incident are shown in Figure 1.

### 2.2. North Sea Area Data Set RSa and RSb (No. 2 and No. 3)

**Figure 2:**
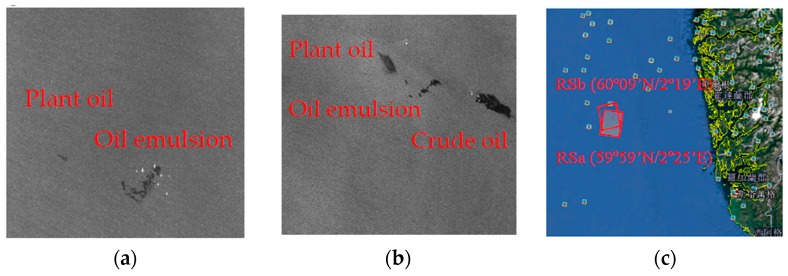
Radarsat-2 scene on 8 June 2011 in the North Sea of Europe. (**a**) Radarsat-2 scene from 8 June 2011, 05.59 UTC (RSa); (**b**) Radarsat-2 scene from 8 June 2011, 17.27 UTC (RSb); (**c**) The location of the incident.

An oil slick experiment performed on 8 June 2011 in the North Sea is shown in Figure 2. Specifically, the Norwegian Clean Seas Association for Operating Companies (NOFO, Sandnes, Norway) conducted this exercise to test equipment and procedures. Plant oil, oil emulsion, and crude oil were released during the exercise. Emulsions of Oseberg blend crude oil mixed with 5% IFO380 was released, and the plant oil here was used to simulate natural monomolecular biogenic slicks, e.g., algae and bacteria, which are often misinterpreted as oil spills in SAR imagery [15]. Oil emulsion and crude oil were recovered and decomposed before SAR acquisitions. Table 2 describes the three oil releases. The left slick in RSa is the plant oil released ~2 h before the acquisition, whereas the remaining part of the emulsion, which was released ~18 h prior to the satellite pass, is seen on the right, see Figure 2a. These slicks are also contained in RSb, with the plant oil slick (~13 h old) to the left, the emulsion (~29 h old) in the middle, and the crude oil (~9 h old) to the right [15], see Figure 2b. The wind speed and wind direction were about 3 m/s and about 150°. The sea water temperature was in the range of 10 °C–17 °C. 

### 2.3. Mexico Bay Area Data Set (No. 4)

**Figure 3:**
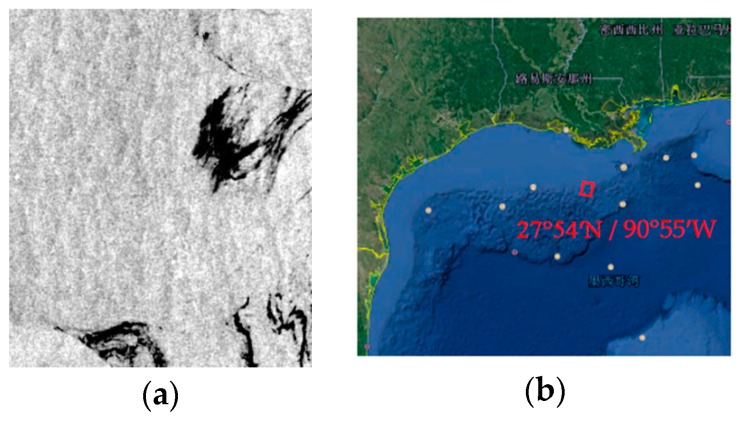
Radarsat-2 scene on 24 August 2011, 12.05 UTC in the Mexico Bay area. (**a**) The gray scale of the oil slick data in the Mexico Bay area; (**b**) The location of the incident.

Radarsat-2 image of the Gulf of Mexico (No. 4) is acquired on 24 August 2011. Explosion of the offshore drilling platform lead to the oil slick. The dark spots in the image have been interpreted as crude oil [40]. On the website (http://www.remss.com/), the wind speed would be above 15 m/s. The gray scale and the location of the incident are shown in Figure 3.

### 2.4. South China Sea Area Data Set (No. 5)

**Figure 4:**
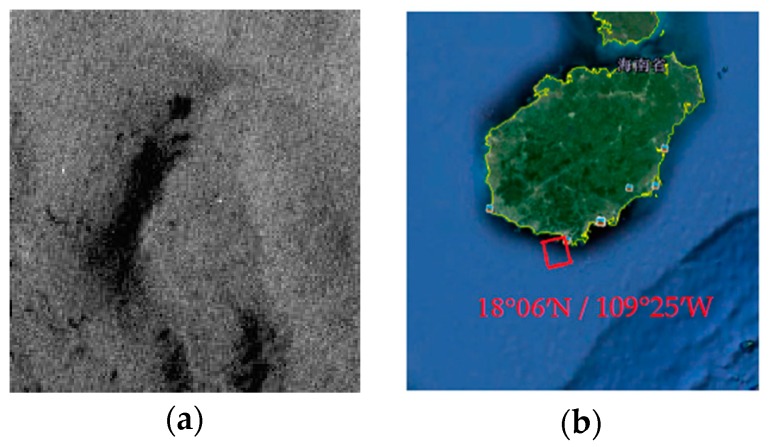
Radarsat-2 scene on 18 September 2009, 10.50 UTC in the South China Sea. (**a**) The gray scale of the oil slick data in the Mexico Bay area; (**b**) The location of the incident.

The data was obtained in the South China Sea on 18 September 2009. The purpose of the experiment in the South China Sea is to make a comparison between crude oils and organic excretions that often revealed as lookalikes [2]. The experimental data contain a small amount of crude oil and plant oil, which were poured with fifteen-minute interval [2]. Plant oil was used to simulate a natural monomolecular biogenic slick. The gray scale and the location of the incident are shown in Figure 4.

## 3. Multi-Feature Discrimination Analysis

### 3.1. Dark Spots Detection

Dark spot detection is the first step in distinguishing oil slicks from lookalikes. In the SAR images, oil slicks and lookalikes appear much darker than surrounding areas. Any region that is darker than its surrounding area should be studied in further detail.

Quad-polarimetric SAR images are susceptible to noise. Pauli decomposition has the advantages of anti-interference and general high adaptability [41]. The Pauli decomposition graph is clearer than original quad-polarimetric SAR graph, and it benefits the detection of dark spots and image post-processing.

Image preprocessing stages are as follows:

1. The original quad-polarimetric SAR data are decomposed by Pauli.
2. The obtained Pauli decomposition graph is filtered by Boxcar filtering.

### 3.2. Feature Extraction

Feature extraction is crucial to the classification of oil slicks and lookalikes. Geometrical (such as *area*, perimeter, and perimeter to area ratio), textural, and polarimetric features are always considered to distinguish oil slicks from lookalikes [1,17,42]. As the input of the proposed classifier, the features discussed in this paper are pixel-based features, which can be selected from feature images one by one. However, geometrical features are region-based features, which are based on adjacent pixel distribution of images. Here, geometric features are not employed in the proposed classification procedure. We extracted 12 features from the first three data sets, including 8 texture features and 4 polarimetric features. The extracted features of the dark spots are shown in Table 3.

#### 3.2.1. Texture Features

The texture features describe the spatial distribution and spatial correlation of gray level. In general, the texture information of oil slicks and lookalikes is different even with similar gray level information. The texture of the oil slicks is continuous, smooth and delicate, while the texture of the lookalikes is scattered, rough, and discontinuous [17]. In this paper, gray level co-occurrence matrix was used to extract texture features.

Because the gray level co-occurrence matrix is a function of angle and offset, all of the texture features calculated using gray level co-occurrence matrix are also functions of angle and offset [11]. For this reason, the choice of angle and offset is the key to texture feature computation. For different images, the best texture features computed by angle and offset are different. The selection of angle and offset used in this paper took place as follows:

- The texture features can be calculated from four angles (0°, 45°, 90° and 135°) in SAR data processing. Offset can be classified as one of three distances (1, 2, and 3).
- The experimental results showed that the 8 texture features of crude oil, plant oil, and oil emulsion samples underwent little change in the 4 different angles.
- During calculation of these three offsets, the texture characteristics of the three kinds of dark spots also changed little.
- In the experiment, the offset and angle of gray level co-occurrence matrix were set to 1° and 45° to extract the texture features.

#### 3.2.2. Polarimetric Features

The polarimetric SAR data have polarimetric information, such as polarimetric matrix and scattering vector, polarimetric data distribution, and target decomposition parameters [1]. Unlike single polarimetric, quad-polarimetric SAR image contains not only intensity information but also phase information. Quad-polarimetric SAR can effectively obtain the scattering characteristics of the target and can more fully reflect the geometry and physical characteristics of the target [1].

In this paper, the polarization scattering entropy (entropy), scattering alpha (alpha), SERD, and Pedestal Height (PH) of three kinds of dark spots (plant oil, emulsion, and crude oil) are extracted. Decomposition of four features is based on an eigenvector decomposition of the (3 × 3) complex coherency [T3] matrix. SERD is expressed as follows:

(1) $$\mathrm{SERD}=\frac{\lambda_{s}-\lambda_{3nos}}{\lambda_{s}+\lambda_{3nos}}$$

where $\lambda_{inos}$ are the eigenvalues of T3, The value of the scattering angle $\alpha_{i}$ can be solved according to the characteristic vector corresponding to the characteristic value $\lambda_{1nos}$ and the characteristic value $\lambda_{2nos}$. If $\alpha_{1}\leq \frac{\pi }{4}$ or $\alpha_{2}\geq \frac{\pi }{4}$, $\lambda_{s}=\lambda_{1nos}$; if $\alpha_{1}\geq \frac{\pi }{4}$ or $\alpha_{2}\leq \frac{\pi }{4}$, $\lambda_{s}=\lambda_{2nos}$. SERD is very sensitive to surface roughness [1]. The value of SERD reflects the proportion of single scattering in the scattering mechanism. The larger the SERD value, the greater the proportion of single scattering in the target scattering mechanism [1]. The oil film suppresses the capillary ripple and short gravity waves on the surface of the ocean. In the low-entropy scattering region, the scattering mechanism is dominated by single scattering, and the SERD value is relatively large. In the high scattering area of the oil film, the scattering mechanism on the surface of the ocean is complex, single scattering is not dominant, and the SERD is smaller. PH is expressed as follows:

(2) $$\mathrm{PH}=\frac{\min\left(\lambda_{1},\lambda_{2},\lambda_{3}\right)}{\max\left(\lambda_{1},\lambda_{2},\lambda_{3}\right)}=\frac{\lambda_{3}}{\lambda_{1}}$$

where $\lambda_{i}$ (*i* = 1, 2, 3) are the eigenvalues of T3. PH is the ratio of the minimum and the maximum eigenvalue, the eigenvalue is related to the optimal backscattering polarization. PH is a measure of the unpolarized component in the average echo [43]. In the area covered by oil slicks, the difference between the minimum and the maximum eigenvalue is not high, and the PH is large.

#### 3.2.3. Feature Analysis and Selection

In order to select the most suitable features, and hence yield the optimum feature subset to discriminate oil slicks from lookalikes, candidate features were analyzed as follows [44]:

- 6 samples of each kind of dark spot on the 12 bands (8 texture feature bands and 4 polarimetric feature bands) were selected.
- The average feature value of all pixels in each sample was calculated.
- The optimal feature subset was decided by analyzing and comparing 12 kinds of feature values of three kinds of dark spots.

**Figure 5:**
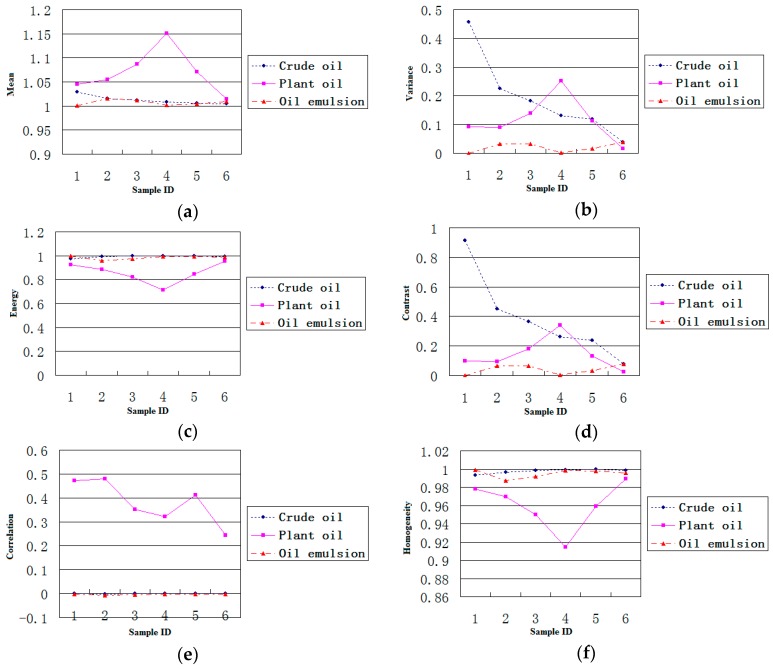

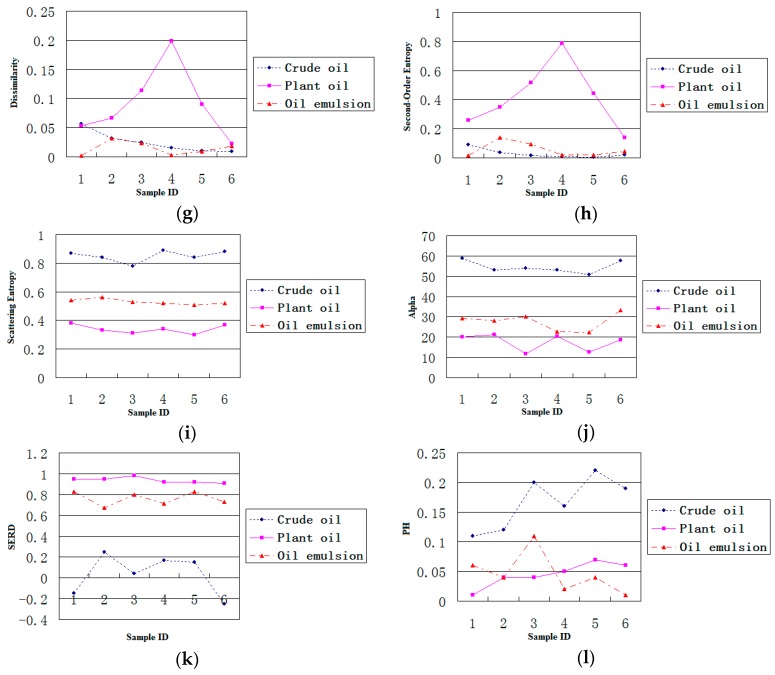
Plots of 12 features values extracted for three kinds of dark spot areas. (**a**) Mean; (**b**) Variance; (**c**) Energy; (**d**) Contrast; (**e**) Correlation; (**f**) Homogeneity; (**g**) Dissimilarity; (**h**) Second-Order Entropy; (**i**) Scattering Entropy; (**j**) Alpha; (**k**) SERD; (**l**) PH.

Figure 5 shows the maps of 12 features extracted for three kinds of dark spots, i.e., crude oil, plant oil and oil emulsion. The experimental results show that three polarimetric characteristics (entropy, alpha, and SERD) can distinguish oil slicks from lookalikes well. Correlation and second-order entropy can distinguish plant oil from the other two kinds of dark spots, but crude oil and emulsified oil films could not be differentiated. PH can distinguish crude oil from other two kinds of dark spots, but plant oil and emulsified oil film could not be differentiated. Through this analysis, scattering entropy, alpha, and SERD were selected for the optimal feature set. 

**Figure 6:**
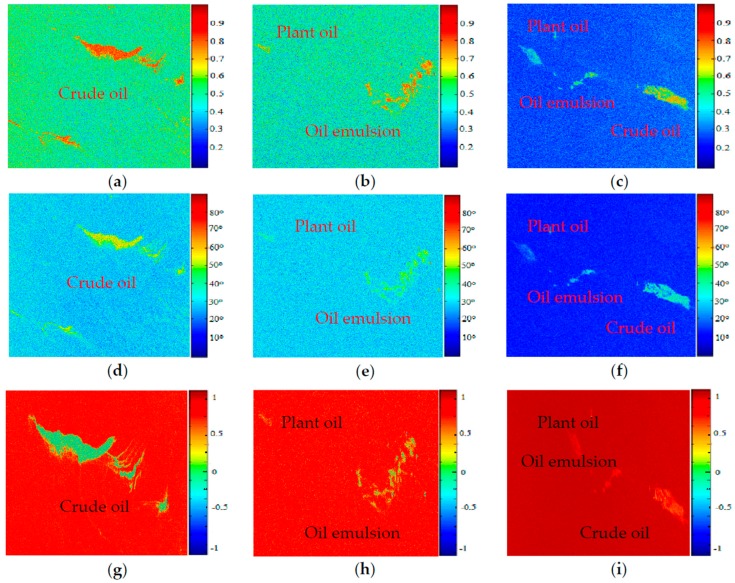
Three kinds of polarimetric feature images of the three data. (**a**) Entropy of Gulf of Mexico oil slick data; (**b**) Entropy of the North Sea of Europe (RSa); (**c**) Entropy of the North Sea of Europe (RSb); (**d**) Alpha of Gulf of Mexico oil slick data; (**e**) Alpha of the North Sea of Europe (RSa); (**f**) Alpha of the North Sea of Europe (RSb); (**g**) SERD of Gulf of Mexico oil slick data; (**h**) SERD of the North Sea of Europe (RSa); (**i**) SERD of the North Sea of Europe (RSb).

The three kinds of polarimetric feature images of the three data were obtained by decomposition and shown in Figure 6. Three ROIs in the same position can be obtained on the three feature images by selecting a ROI. Through K-fold Cross Validation (seeing Section 4.4.3 for more details), 6300 ROIs were chosen and divided into 5400 training sets and 900 test sets. The training data sets consist of 1800 crude oil, 1800 plant oil and 1800 oil emulsion samples. Each training data set was equally divided into three groups for scattering entropy, alpha and SERD parameters. The distribution rules of the 900 test data sets are similar to that of training data sets. The size of each ROI was 28 × 28 (seeing Section 4.4.1 for more details), the value of each pixel corresponded to the feature value of the three polarization features rather than the gray value.

#### 3.2.4. Parameter Analysis of Boxcar Filter

Data to be decomposed were run through an additional filtering procedure, a Boxcar filter. A sliding window of N × N dimensions was used to compute the local estimate of the average matrix. If the window size of Boxcar filter was set to 1, the process of data decomposition would avoid any need for additional filtering. Filtering can significantly improve the accuracy of supervised classification. The Boxcar filter is better than the refined Lee filter in improving classification accuracy [37]. The size of the filter window also exerts an important influence on the classification accuracy.

**Figure 7:**
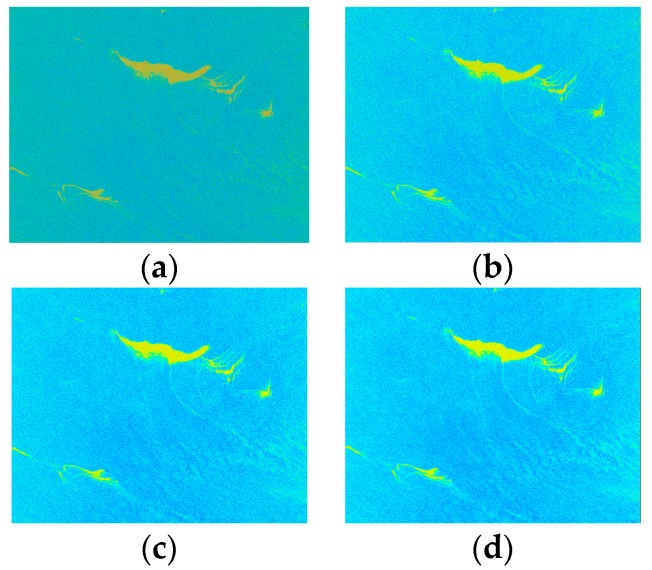
The feature images of alpha of the data of the Gulf of Mexico. (**a**) alpha_window size 1 (without boxcar filtering); (**b**) alpha_window size 3; (**c**) alpha_window size 5; (**d**) alpha_window size 7.

**Figure 8:**
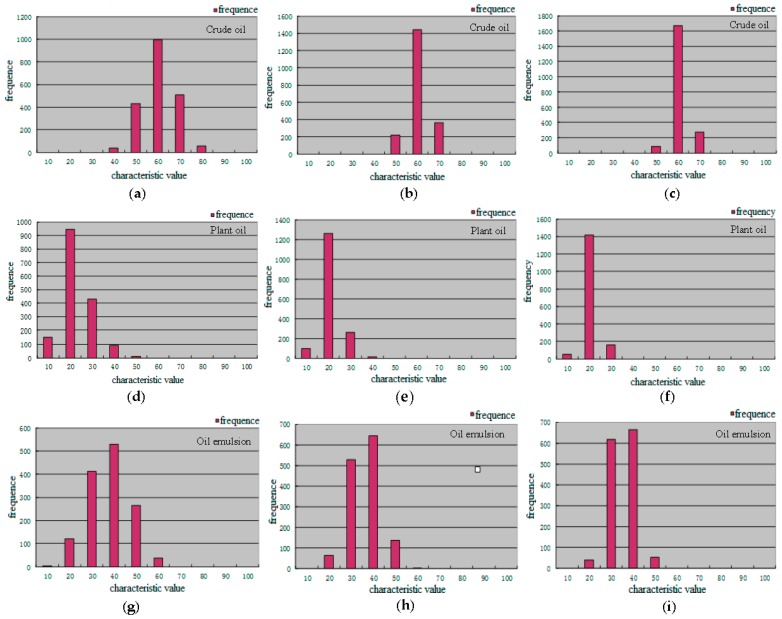
Polarized scattering alpha distribution of the selected region. (**a**) Window size 3 × 3 (Crude oil); (**b**) Window size 5 × 5(Crude oil); (**c**) Window size 7 × 7 (Crude oil); (**d**) Window size 3 × 3 (Plant oil); (**e**) Window size 5 × 5 (Plant oil); (**f**) Window size 7 × 7 (Plant oil); (**g**) Window size 3 × 3 (Oil emulsion); (**h**) Window size 5 × 5 (Oil emulsion); (**i**) Window size 7 × 7 (Oil emulsion).

In order to determine the size of the Boxcar filter window, we selected one area containing each kind of dark spots (crude oil, plant oil, and oil emulsion) in three data sets; hence we set the boxcar filter window size to 3 × 3, 5 × 5, and 7 × 7. The polarimetric features of selected regions on the three polarimetric feature images were compared under the different window sizes. Taking the polarized scattering alpha as an example, we found that the expansion of the filter window cause the edges become more blurred. The Edge Preserving Index (EPI) in Table 4 illustrated this point. Some of the sea areas become brighter, as shown in Figure 7, and the feature value of sea areas and dark spots became larger. The number of pixels with large feature values also increased with the expansion of the filter window, as shown in Figure 8. Because filtering improves the classification accuracy, we did not consider the case when the window size was set to 1, as shown in Figure 8 (without Boxcar filtering). This phenomenon may lead to misjudgment. The filtering time also increased significantly with window size, so we set the window size to 3.

## 4. Oil Slick Classification Based on CNN

### 4.1. Introduction to CNN

CNN is a feed-forward neural network, and it has excellent performance for image recognition [45]. CNN is a multilayer perception which is designed to recognize two-dimensional shapes [45]. The basic structure of CNN consists of two layers: In the feature extraction layer, the input of each neuron is connected to the Local Receptive Fields (LRF) of the previous layer; the other is the feature mapping layer [45]. Each layer of the computing network is composed of a number of feature maps, and each feature map covers a plane, and all neurons on that plane have equal weight. The LRF and Weight-Sharing (WS) of the CNN structure can greatly reduce the number of parameters of the network structure and accelerate training. The network structure is highly invariant to translation, scaling, and inclination. The WS network structure of neural network reduces the complexity of network model and reduces the amount of weight. This advantage is more obvious when the input of the network is a multi-dimensional image, so the image can be directly used as the input of the network, and it avoids any need for the complex feature extraction and data reconstruction processes required by the traditional recognition algorithm [26,27,28,29,30,31,32,45]. CNN mainly includes alternating convolutional layer and pool layer [45].

The input image is convoluted by trainable filters and an additive bias. After convolution, feature maps are generated in the C1 layer, four pixels from each group on the feature map are then weighted and added together, hence S2 layers were obtained using a sigmoid function. These images are then filtered to find the C3 layer. S4 is produced by C3 through the same process by which S2 is produced by C1. Finally, these pixel values are rasterized and connected into a vector as the input of the traditional neural network to get the output [45].

The C layer is the feature extraction layer. The input of each neuron is connected to the LRF of the previous layer, and then the local features are extracted. Once these local features are extracted, the relationship between its position and that of other features is also determined. The S layer is a feature mapping layer, and each layer of the network is composed of multiple feature maps. Each feature is mapped into a plane, and the weights of all neurons on the plane are equal. The sigmoid function serves as the activation function of the CNN, so feature mapping has the invariance of the displacement [45].

The training process of CNN is divided into four steps:

1. A sample (X, YP) from the sample set is taken and X is entered into the net.
2. The actual output (Op) is calculated.
3. The difference between Op and the corresponding ideal output YP is calculated.
4. The weight matrix of the back propagation method is adjusted based on the minimum error.

In steps 1 and 2, the information is transferred from the input to the output layer through gradual transformation. (The final results are obtained using the multiplication of the input and the weight matrix of each layer.) The classification results are obtained using the trained network structure.

### 4.2. Classification of Oil Slicks and Lookalikes Based on CNN

**Figure 9:**
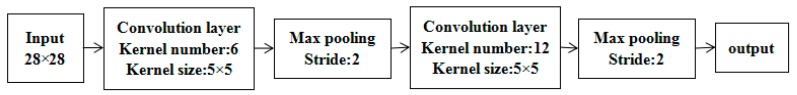
The structure and parameters of CNN.

**Figure 10:**
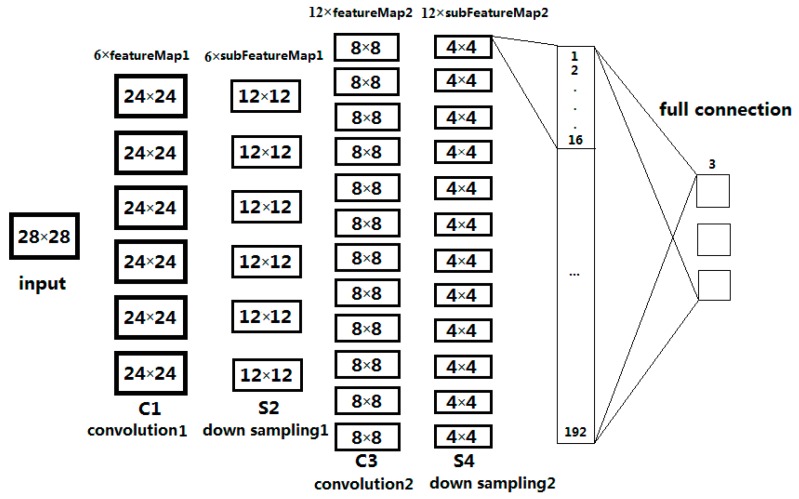
CNN structure used in the experiment.

**Figure 11:**
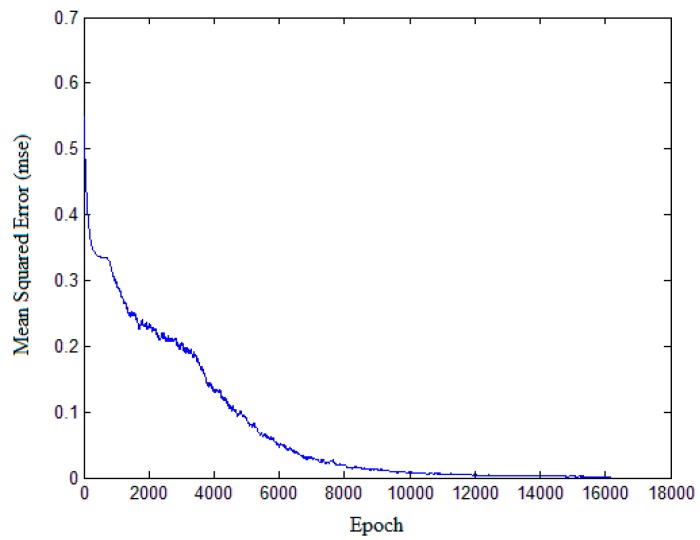
CNN training performance.

The three polarimetric features in the previous section here serve as the basis for classification of oil slicks and lookalikes. The learning process of the network is based on the gray value of all pixels of the input image in original CNN. However, in this paper, the learning process of network is based on feature values. Each input sample is selected from the feature images. A total of 6300 samples were selected from the three kinds of feature images, including 2100 crude oil samples, 2100 plant oil samples, and 2100 oil emulsion samples. The size of ROI (input sample) is 28 × 28, and the parameter will be analyzed in Section 4.4.1. CNN is applied to a training data set of 5400 samples, including 1800 crude oil, 1800 plant oil, and 1800 oil emulsion samples. The structure and parameters are given in Figure 9, according to the original version of CNN [32]. The CNN structure used in the experiment is shown in Figure 10. The classification accuracy determined by using the test data set of 900 samples (equally divided among crude, plant and emulsion oil samples) is 91.33%. The result of classification based on multi-features fusion is shown in Table 5, and the training performance is shown in Figure 11. In order to obtain more reliable results statistically and avoid overfitting, a K-fold Cross Validation (K-CV) was be implemented in detail (seeing Section 4.4.3).

### 4.3. Comparison of CNN to ANN

We performed another experiment in order to draw a comparison between the introduced algorithm and ANN.

The input of CNN is sample images selected on the feature images, but the input of ANN is feature vectors composed of three kinds of polarimetric features. Selecting a sample on the feature image can produce three feature samples at the same position (entropy, alpha, and SERD feature samples), so 5400 training samples in CNN correspond to the 1800 training samples in ANN, and 900 test samples in CNN correspond to the 300 testing samples in ANN. Then, the mean value of all the feature sample pixels is calculated as the feature value of the sample. In this way, we get three feature values for the three kinds of feature samples in the same position, which can be composed to a feature vector and taken as an input sample of ANN. So, the ANN training data set has 1800 samples (equally divided among crude, plant and emulsion oil samples); the test data set has 300 samples (equally divided among crude, plant and emulsion oil samples).

**Figure 12:**
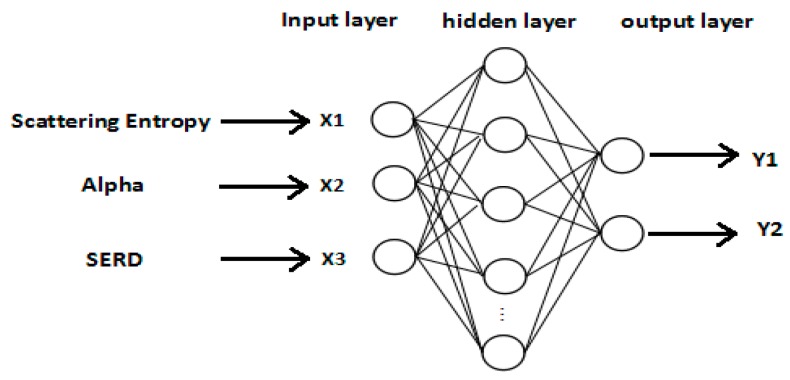
Network structure of ANN.

The network structure of ANN is shown in Figure 12. Input layer nodes X1–X3 represent polarization scattering entropy (entropy), scattering alpha (alpha), and SERD. The output layer has two nodes, the values can be either 0 or 1. The output representation is shown in Table 6.

According to the general design principle, the transfer function of the hidden layer neuron is logsig. The transfer function of the output layer neuron is purelin. According to the Kolmogorov theorem, the formula of hidden layer node number set is $l=\sqrt{m+n}+a$, where “*m*” is the number of input nodes, “*n*” is the number of output nodes, and “*a*” is the constant of 1–10 [46]. According to the ANN structure in this paper, the number of nodes in the input layer is 3, and the number of nodes in the output layer is 2, so the number of nodes in the hidden layer ranges from 4 to 13. There are three kinds of ANN training function: traingdx, trainlm, and traingd. The experiment shows that training of trainlm takes place over fitting, and the training of traingd takes place under fitting. For this reason, traingdx was adopted as the training function, and the experiment showed that the network error is the smallest when there were 10 nodes in hidden layer. Table 7 is the final network structure.

**Figure 13:**
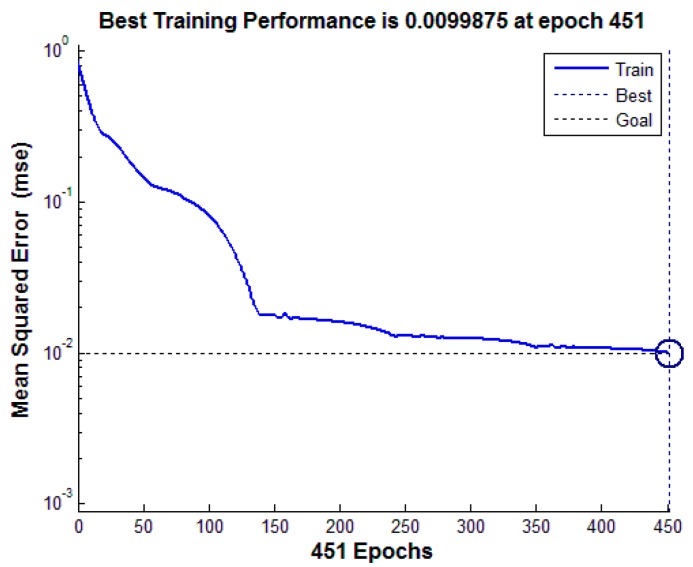
ANN network training performance.

The results showed that 10 samples of oil emulsion were misidentified as crude oil, 15 samples of oil emulsion were mistaken for plant oil, 17 samples of crude oil were mistaken for oil emulsion, and 17 samples of plant oil were mistaken for oil emulsion. In this study, the classification accuracy of ANN is 80.33% with 300 test data sets. The performance of the ANN network training is shown in Figure 13. The results of classification based on ANN with multi-features fusion are shown in Table 8. LRF and WS are two major advantages of CNN. These two advantages reduce the number of weights, simply the neural network structure, and the image can be directly input to the network. The experimental results show that the recognition accuracy of the proposed algorithm is better than that of ANN. The comparison of the proposed algorithm and the ANN method is shown in Table 9. It is found that overfitting phenomena does exist when the training data set is insufficient, which will be analyzed in Section 4.4.2.

**Figure 14:**
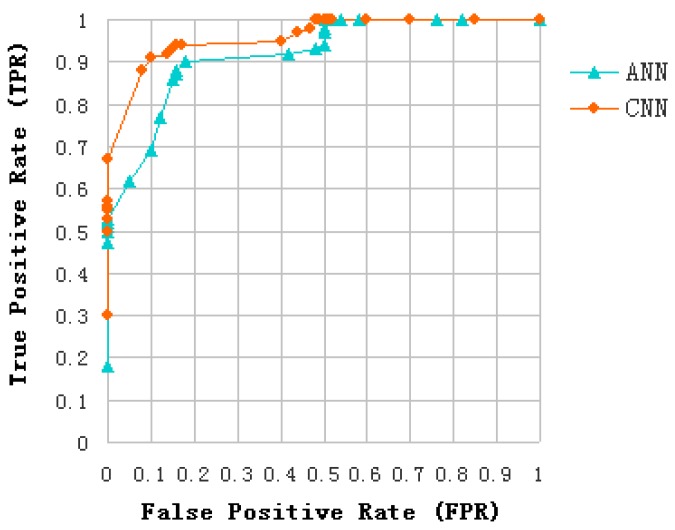
ROC curve of CNN and ANN.

Since the proposed problem can be seen as a detection problem, i.e., discrimination between oils slicks and lookalikes (plant oil and oil emulsion), the estimation of Receiver Operating Characteristics (ROC) curves is used to evaluate and compare the classification effect of the introduced algorithm and ANN. In order to guarantee the probability of oil slicks and lookalikes is equal, the proposed method is applied to a training data set of 3600 samples, including 1800 oil slick and 1800 lookalike samples (equally divided among plant oil and oil emulsion samples). The distribution rules of the 600 test data sets are similar to that of training data sets. According to the correspondence between the CNN samples and the ANN samples (as introduced in Section 4.4), ANN training data set has 1200 samples and test data set has 200 samples, and the distribution rules of the samples are similar to that of CNN. The ROC curve of CNN and ANN are shown in Figure 14. Some results can be analyzed as follows:

- Area Under Curve (AUC) of CNN and ANN were over 90%, and the AUC of CNN is greater than that of ANN. This indicates that both of them have good separability in distinguishing oil slicks and lookalikes.
- The position of the Equal Error Rate (EER) of CNN is at False Positive Rate (FPR) = 0.09, and the position of the EER of ANN is at FPR = 0.15. It can be seen that the CNN model is more suitable for the distinction between oil slicks and lookalikes than ANN.

### 4.4. Parameter Analysis

#### 4.4.1. Size of ROI

The size of ROI (input sample) affects the classification of CNN. If the input patch is too small, CNN will be unable to fully learn the features of the image. If it is too large, too heavy burden is placed on the network, then training and testing become too time-consuming. Therefore, it is important to determine the size of input patch for the classification of CNN. To this purpose, we performed experiments to show the effect of different input sizes on the classification results considering input samples of 20 × 20, 24 × 24, and 28 × 28, see Table 10. As shown, the consistency of the classification increases with the increase of the input samples. We can see that the accuracy increases slowly after the sample size of 24 × 24, which means the classification accuracy tends to remain stable. Larger samples will increase the burden on the network and require longer testing and training periods. For this reason, we set the size of the input patch to 28 × 28.

#### 4.4.2. Overfitting Analysis

Two experiments were implemented in order to test whether overfitting existed in the proposed algorithm when the training data set is insufficient. Here, the probability of each kind of oil slicks and lookalikes is equal. The first model was trained based on the first three data (No. 1~No. 3), including 5400 samples (1800 crude oil, 1800 plant oil, and 1800 oil emulsion samples). The second trained model was only based on the third data (No. 3), including 2700 samples (900 crude oil, 900 plant oil, and 900 oil emulsion samples). These two trained models were used to test the same test data, which selected from the third data (No. 3), including 450 samples (150 crude oil, 150 plant oil, and 150 oil emulsion samples). According to the correspondence between the CNN samples and the ANN samples (as introduced in Section 4.4), ANN training data of the first and second model has 1800 samples and 900 samples respectively, test data has 150 samples, and the distribution rules of the samples are similar to that of CNN. Classification accuracy of each kind of oil slicks and lookalikes based on the first and second model are shown in Table 11. On Table 11, the classification accuracy of the second model is higher than that of the first model. This proves overfitting phenomena does exist when the training sample space is insufficient. It’s necessary for us to enlarge training sample size to avoid overfitting.

#### 4.4.3. K-Fold Cross Validation

In order to obtain more reliable results statistically, K-CV has been implemented. K-CV can effectively avoid overfitting and underfitting, and the final results are more persuasive [47]. Several partitions of the data set should be tested in order to check the validity of the trained models. Thus, the data set was divided into K groups randomly. Each subset of data set was used as a test set respectively, the rest of the data set (K-1 groups) was used as the training set. Hence, the selection of the K value is critical. The average and variance of classification accuracy was used to validate the performance based on the K-CV. The results with the K = 3, 5, 7 and 9 are shown in Table 12. We can see that the trained model is valid, and the classification constancy is improved by increasing K. The average and variance of classification accuracy increase slowly after the K value of 7. Taking into account both statistical stability and computational cost, the value of K is set to 7. In this paper, 6300 samples were divided into two parts: 5400 samples (training data) and 900 samples (test data).

### 4.5. Classification Experiments and Analysis

The performances of the proposed approach are evaluated on the whole experimental SAR dataset. Firstly, we used the previously trained CNN model to test the dark spots of the first three sets (No. 1~No. 3) of data respectively, which contain both training and test data when established CNN model. Then, the dark spots on the scene No. 4 and No. 5 (only used for the prediction) were predicted to be oil slicks or lookalikes. Here, the proportions of each kind of oil slicks and lookalikes are equal prior probability. The classification accuracies are shown in Table 13. Moreover, analyses were conducted as follow:

(1) The proposed method can distinguish oil slicks and lookalikes effectively, and its classification accuracy is higher than that experienced with the ANN approach. These results are consistent with the analysis ROC curve (as discussed in Section 4.3).
(2) The classification accuracy results in Table 13 show high variance. The first three data (No. 1~No. 3) were used to build the CNN model, and their classification accuracy is higher. However, the last two experimental data (No. 4, No. 5) were used only for prediction, which is not including any training data, and their classification accuracy is much lower. If the provided data is more sufficient then their classification accuracy would be much better than we expected. On the other side, overfitting phenomenon does exist when the provided data set is insufficient. Therefore, it is necessary to enlarge data set to avoid overfitting and make the CNN model to be more stabilized.
(3) The scene No. 4 and No. 1 are from the same sea area (Gulf of Mexico), but testing accuracy of crude oil on the scene No. 4 is lower than that on the scene No. 1. This indicates that the recognition rate of the proposed method will be affected by the sea water temperature and wind speed. This is because the scene No. 4 was captured in August 2011, when the sea water temperature of the Gulf of Mexico reaches its highest level (28 °C), while it is about 20 °C in May. Moreover, the wind speed is greater than that of scene No. 1, see the ripples in scene No. 4.
(4) It can be seen from the classification result of scene No. 5 that sea water temperature, sea area conditions, and climatic conditions will affect the classification accuracy of the proposed approach. Training data from the Gulf of Mexico and North Sea, South China Sea is far from the two sea areas, and the sea water temperature, environmental and climate conditions are differ from those of the two sea areas.

## 5. Conclusions and Outlooks

The current study is to discriminate oil slicks from lookalikes in polarimetric SAR images based on the use of multi-feature fusion and a proposed CNN approach. It allows exploiting the information content of polarimetric features obtained from the decomposition of quad-polarimetric SAR images, ensuring a high classification rate to distinguish oil from lookalike slicks. Effectively compared with respect to a classical ANN approach, the proposed method is able to identify accurately the dark spots on SAR images and hence to classify unstructured features related to different oil classes.

In consideration of the different contributions of each polarimetric feature to the classification performance, a simple discrimination analysis was conducted to assess discrimination ability of each feature. We have explored 12 features of oil slicks and lookalikes for the purpose of oil slick discrimination, and find a preferred features subset includes entropy, alpha, and SERD in the C-band polarimetric mode. 

A solution for multi-feature fusion of the three polarimetric features is provided with the proposed CNN model. In order to avoid overfitting and obtain more reliable results statistically, we extend the training data set and a K-CV was be implemented. In the process of CNN model establishment, a training data set consists of 5400 samples, and the classification accuracy was 91.33% with a test data set of 900 samples. 

The effectiveness of the algorithm is demonstrated through the analysis of the five experimental data. These data contain multi-temporal quad-polarimetric Radarsat-2 SAR information of oil slicks and lookalikes (oil emulsion and plant oil). The plant oil in data set is used to simulate a natural monomolecular biogenic slick. The results of the discrimination process on real SAR images demonstrated that the proposed method is not only accurately identify dark spots on SAR images but also classify unstructured features.

This study gives an important method to distinguish oil slicks from lookalikes, and it is effective for oil slicks classification task. The result also shows if the difference of sea condition (such as climatic, geographical, sea temperature and environmental conditions) between test and training data is too large, classification accuracy would be a big decline. In order to reduce the effects of these factors on future experiments, we will apply more oil slick data from different sea areas to build the CNN model, and enhance the robustness of training structure of the proposed method. If the prior probabilities of oil slicks and lookalikes are taken into account for classification decision, the classification accuracy would be improved undoubtedly. However, there are many types of lookalikes, and only plant oil and oil emulsion are discussed in this study. The five images used do not contain typical lookalikes caused by low wind or biogenic materials, which are also regarded as major challenges in oil slicks detection. It is very difficult to obtain the priori probabilities of all oil slicks and lookalikes accurately. Therefore, the method of equal probability sampling is adopted in this paper. With the abundance of data sets, prior probability should be used to estimate classification results.

In our study multi-feature fusion, which is also known as early fusion, is implemented and a single complex classifier (CNN) is used. Fusion at different stages of classification procedures is a booming research field that has shown capabilities for improvement of classification results. Late fusion of scores of several classifiers will also be adapted to the proposed problem as a future research work.

## Figures and Tables

**Table 1:** Information of polarimetric SAR sets.

Scene ID	Location of Image Center	UTC	Mode	Incidence Angles	Wind Speed
No. 1	26°49′ N/92°01′ W	2010-05-08 12:01:25.48	Fine Quad	42.10° (average)	~6.5 m/s
No. 2	59°59′ N/2°25′ E	2011-06-08 05:59:34.78	Fine Quad	46.20° (average)	1.6–3.3 m/s
No. 3	60°09′ N/2°19′ E	2011-06-08 17:27:53.33	Fine Quad	34.62° (average)	1.6–3.3 m/s
No. 4	27°54′ N/90°55′ W	2011-8-24 12:05:27.55	Fine Quad	31.50°(average)	~15 m/s
No. 5	18°06′ N/109°25′ E	2009-9-18 10:49:35.42	Fine Quad	32.56°(average)	~10 m/s

**Table 2:** Information of the three oil releases.

	Plant Oil	Emulsion	Crude Oil
Date (release time)	8 June 2011 (04.10)	7 June 2011 (12.15)	8 June 2011 (08.23)
Volume	0.4 m^3^	20 m^3^	30 m^3^
Subject to	Untouched slick	Mechanical recovery (~1 m^3^ left on surface)	Dispersion (on-going)

**Table 3:** The extracted features.

Texture Features	Polarimetric Features
Mean	Scattering Entropy
Variance	Alpha
Energy	SERD
Contrast	Pedestal Height (PH)
Correlation	
Homogeneity	
Dissimilarity	
Second-Order Entropy	

**Table 4:** EPI with different window sizes.

Window Size	EPI
3 × 3	0.2474
5 × 5	0.1213
7 × 7	0.0750

**Table 5:** The result of classification based on multi-features fusion with the input patch is 28 × 28 and κ = 0.87.

Types	Crude Oil (%)	Plant oil (%)	Oil Emulsion (%)
**Crude oil**	92.67	0	11.00
**Plant oil**	0	92.33	0
**Oil emulsion**	7.33	7.67	89.00
**Total**	100	100	100

Note: The accuracy (%) is 0–20% as slight, 21–40% as fair, 41–60% as moderate, 61–80% as substantial, and 81–100% as perfect.

**Table 6:** Output Representation.

Y1 Y2	Representation
1 0	Crude oil
0 1	Plant oil
1 1	Oil emulsion

**Table 7:** Final structure of ANN.

ANN Network Structure	Parameter
Network structure	Single hidden layer
Number of neurons in hidden layer	10
Training function	Traingdx
Network error	0.4872

**Table 8:** The result of classification based on ANN with multi-features fusion and κ = 0.7050.

Types	Crude Oil (%)	Plant Oil (%)	Oil Emulsion (%)
**Crude oil**	83.00	0	10.00
**Plant oil**	0	83.00	15.00
**Oil emulsion**	17.00	17.00	75.00
**Total**	100	100	100

Note: The accuracy (%) is 0–20% as slight, 21–40% as fair, 41–60% as moderate, 61–80% as substantial, and 81–100% as perfect.

**Table 9:** Comparison of the proposed algorithm and ANN.

Classification Method	Classification Accuracy
The proposed algorithm	91.33%
ANN	80.33%

**Table 10:** Accuracy and kappa coefficients for the three feature image with different sizes of the input image sample.

Size	Accuracy (%)	Kappa
20 × 20	86.89	0.8034
24 × 24	90.89	0.8633
28 × 28	91.33	0.8700

**Table 11:** Classification accuracy based on the first and second model.

Training Data	Crude Oil		Plant Oil		Oil Emulsion	
	CNN	ANN	CNN	ANN	CNN	ANN
No. 1~No. 3	96.00%	88%	97.33%	86%	96.67%	88%
No. 3	98.66%	92%	99.33%	88%	98.66%	90%

**Table 12:** The average and variance of classification accuracy based on the K-CV.

K	Average	Variance
3	0.8717	0.0015
5	0.9079	0.0008
7	0.9308	0.0004
9	0.9356	0.0004

**Table 13:** Classification accuracy.

Scene ID	Crude Oil		Plant Oil		Oil Emulsion	
	CNN	ANN	CNN	ANN	CNN	ANN
No. 1	98.67%	92%	-	-	-	-
No. 2	-	-	94.67%	84%	96.00%	86%
No. 3	96.00%	88%	97.33%	86%	96.67%	88%
No. 4	82.67%	72%	-	-	-	-
No. 5	71.33%	62%	72.66%	66%	-	-
